# Assessment of iron bioavailability from different bread making processes using an *in vitro* intestinal cell model

**DOI:** 10.1016/j.foodchem.2017.01.130

**Published:** 2017-08-01

**Authors:** I. Rodriguez-Ramiro, C.A. Brearley, S.F.A. Bruggraber, A. Perfecto, P. Shewry, S. Fairweather-Tait

**Affiliations:** a Norwich Medical School, University of East Anglia, Norwich, UK; b School of Biological Sciences, University of East Anglia, Norwich, UK; c MRC Human Nutrition Research, Cambridge, UK; d Rothamsted Research, Hertfordshire, UK

**Keywords:** Iron uptake, Myo-inositol hexa*kis*phosphate, Sourdough bread, Caco-2 cells, Simulated gastrointestinal digestion

## Abstract

Myo-inositol hexa*kis*phosphate (IP6), is the main iron chelator in cereals and bread. The aim of this study was to investigate the effect of three commercial baking processes (sourdough, conventional yeast and Chorleywood Bread Making Process (CBP)) on the IP6 content of wholemeal bread, its impact on iron uptake in Caco-2 cells and the predicted bioavailability of iron from these breads with added iron, simulating a mixed-meal. The sourdough process fully degraded IP6 whilst the CBP and conventional processes reduced it by 75% compared with wholemeal flour. The iron released in solution after a simulated digestion was 8-fold higher in sourdough bread than with others but no difference in cellular iron uptake was observed. Additionally, when iron was added to the different breads digestions only sourdough bread elicited a significant ferritin response in Caco-2 cells (4.8-fold compared to the other breads) suggesting that sourdough bread could contribute towards improved iron nutrition.

## Highlights

• Fe availability in three commercial bread baking process was compared.
• The sourdough bread baking process fully degraded phytic acid, a main Fe chelator.
• More Fe was released by simulated digestion from sourdough bread.
• The highest Fe uptake was achieved with sourdough bread in a simulated mixed-meal.
• The sourdough bread process could be more beneficial for iron nutrition.

## 1 Introduction

Iron deficiency is one of the most common nutritional deficiencies in humans and is responsible for at least 50% of the 2 billion cases of anaemia worldwide (Lopez et al., 2016, Zimmermann and Hurrell, 2007). Iron deficiency anaemia affects maternal and child morbidity and mortality, decreases physical performance and increases the burden of the health care in developing and developed countries (Kassebaum et al., 2014, Lopez et al., 2016). Iron deficiency anaemia is therefore a public health concern and nutritional intervention approaches are required to ameliorate its impact.

Interest in vegetarian diets has increased in recent decades because of the proposed health benefits associated with plant-based diets, together with concerns over sustainability and affordability (Gibson, Heath, & Szymlek-Gay, 2014). However, in plant-based diets, iron exists in the form of inorganic non-haem iron, which is less bioavailable than haem iron from animal foods sources, and this results in lower total iron absorption from vegetarian diets (Hurrell & Egli, 2010). Cereals, and in particular wheat and bread, are among the most widely consumed staple foods and are of global importance to human nutrition. However, most of the iron in wholewheat flour is present as phytates, salts of phytic acid (myo-inositol hexa*kis*phosphate (IP6)) which reduce iron bioavailability during the intestinal transit (Davidsson, 2003, Hurrell and Egli, 2010). Different strategies have been employed in order to increase bioavailable iron in cereal products, including supplementation, food fortification, biofortification (via plant agronomy, breeding or genetic engineering) and changing dietary habits through consumer education (Abbaspour et al., 2014, Zimmermann and Hurrell, 2007). Additionally, in order to counteract the negative effect of phytate, the use of exogenous phytase enzymes and different bread making processes have been explored (Baye et al., 2015, Brune et al., 1992, Lopez et al., 2001, Sanz-Penella et al., 2009). In particular, the use of lactic acid bacteria in sourdough bread production has shown a significant degradation of IP6 (Arendt et al., 2007, Haros et al., 2007, Palacios et al., 2008, Sanz-Penella et al., 2009), suggesting that modifications in the processes of fermentation and baking (i.e. temperature, leavening time and microorganism strain) could improve iron bioavailability. It is therefore important to determine how these different technologies affect iron absorption from bread at the intestinal level. However, human intervention trials are lengthy and expensive, which restricts their application when evaluating strategies to reduce iron deficiency. Consequently, the combined *in vitro* digestion/Caco-2 intestinal cell model has been extensively used to predict iron bioavailability from food sources and to investigate the intestinal cellular mechanisms of iron uptake (Fairweather-Tait et al., 2005, Thompson et al., 2010). For example, Eagling, Wawer, Shewry, Zhao, and Fairweather-Tait (2014) compared differences in iron bioavailability between unleavened breads made from wholegrain and white flours from two commercial wheat cultivars and reported an inverse relationship between the cellular iron uptake and phytate content. Similarly, Sanz-Penella, Laparra, Sanz, and Haros (2012) have investigated the use of a *Bifidobacterium* strain, as a starter in the baking process, to increase the available iron through phytate degradation. Their findings suggest that either longer fermentation times or the inclusion of more active phytases are required to completely abolish the phytate content thereby leading to improvement in iron bioavailability from bread. The aims of the present study were therefore i) to compare the effects of three commercial processes of bread making on the reduction of phytate in wholemeal bread, ii) study the resulting impact on iron bioavailability using an intestinal cellular model, and iii) to predict how the different bread matrices might affect cellular uptake of iron from exogenous sources by adding ferrous sulphate to simulate the contribution of non-haem iron from a mixed-meal.

## 2 Material and methods

### 2.1 Reagents

Chemicals, enzymes and hormones were purchased from Sigma-Aldrich, UK unless otherwise stated.

### 2.2 Grain material and bread making process

Grain (20 kg), from the commercial bread wheat cultivar Hereward, grown in the field of Rothamsted Research, UK, in 2014, was milled using a Buhler−MLU-202 mill at Campden BRI according to TES-CM-01. This resulted in 10 fractions (Table 1), including six white flour fractions: three breaks (B1-B3) and three reductions (R1-R3). These fractions varied in purity, with the first break (B1) and reduction (R1) corresponding to the purest starchy endosperm tissue and the third break (B3) and reduction (R3) to the least pure. The remaining four fractions consisting of bran fractions which were separated into offal flour, bran flour coarse offal and coarse bran. The 10 fractions were recombined and the resultant wholemeal flour was used to make three different breads based on commercial processes: Chorleywood bread process (CBP), conventional, and sourdough bread-making processes. The ingredients for each process are described in Table 2. For the CBP, the dough was mixed and proved for 60 min at 30 °C. The conventional and the sourdough processes followed the two-step sponge and dough method. For the sourdough process, a commercial starter culture (Lesaffre LV1) was mixed with flour, water and salt for 5 min and fermented at 28 °C for 24 h. Additional flour, yeast, water, salt and fat were then added and the sample mixed for 6 min, bulk fermented for 4 h at room temperature, and proved for 5 h at 25 °C. For the conventional system, yeasted dough was mixed for 5 min and fermented 16 h at 14 °C, additional flour, yeast, water, salt and fat were added, mixed again for 10 min, bulk fermented at room temperature for 15 min and proved at 30 °C for one hour. All breads were baked at 230 °C for 30 min. The breads were sliced and stored frozen at −80 °C until used. The crusts of the slices were then removed and the crumb was freeze-dried, finely ground and stored in a desiccator at 4 °C over silica gel.

### 2.3 Determination of iron in the bread and simulated digestion samples

The concentrations of Fe were determined using an Inductively Coupled Plasma Optical Emission Spectroscopy (ICP-OES, Jobin Yvon Horiba – ULTIMA 2C) equipped with a glass concentric nebulizer (1 ml/min sample flow rate), a 50 ml glass cyclonic spray chamber and a radial torch with a 3 mm i.d. alumina injector. Sample solutions were introduced from an auto-sampler (Jobin Yvon Horiba AS500) using a sample probe with 0.5 mm i.d. sample tubing and 0.76 mm i.d. pump tubing (black/black). Instrument operating conditions are listed in Supplementary Table 1. Peak profiles were used to measure Fe as described in Supplementary Table 2.

The dry ground breads (about 500 mg) were weighed directly into 15 ml TFM® PTFE microwave vials, to which 3 ml of UHP water and 3 ml of 69% HNO_3_ were successively added. The vials were placed into a microwave digestion system (UltraWAVE, Analytix, UK) and heated at 230 °C for 15 min. The digested solutions were decanted into pre-weighted 15 ml PP tubes and the microwave vials rinsed twice with 3 ml of UHP water which was then added to the decanted digestates. The tubes were weighed to determine the total mass of digestates. The digestates were then diluted in water to a final acid concentration of 5%. Digestion blank controls and internal quality controls were prepared alongside the food samples and analysed with the digested samples. A series of external calibration standards containing Fe were prepared from commercial standard stock solutions (1000 ± 2 ppm in 2% HNO_3_; PerkinElmer Pure Plus), with final concentrations ranging from 0 to 1 ppm in a diluent matched to the digested solution (final concentration of 5% HNO_3_). The Fe concentrations were calculated against the linear regression obtained from the calibration standard curve performed with each analysis (Supplementary Fig. 1). The flour fractions used to make the wholemeal bread were analysed by ICP-OES following a similar procedure.

For analysis of the simulated gastrointestinal digestions, the instrument was equipped with a concentric PFA micro-flow nebulizer (0–2 ml/min sample flow rate), a 50 ml glass cyclonic spray chamber and a radial torch with a 3 mm i.d. alumina injector. Sample solutions were introduced from an auto-sampler (Jobin Yvon Horiba AS500) using a sample probe with 0.25 mm i.d. sample tubing and 0.38 mm i.d. pump tubing (orange/green). The instrument running conditions were as described in Supplementary Table 3 and the peak profile measurement parameters were as in Supplementary Table 2.

The simulated gastrointestinal digestions of the bread (400 μl) were placed into (2 ml) cryovials and 800 μl of fresh solution (30%) H_2_O_2_/(69%) HNO_3_ (1:1, v/v) were added and incubated at room temperature overnight and then at 40 °C with the cap unscrewed. The samples were then diluted in water to 5% HNO_3_ concentration and the Fe concentrations calculated.

The fractions of bioaccessible and precipitated iron were determined using the following Eqs. (1), (2), respectively:

(1) $$[(\%)\text{Bioaccessible Fe}]=[({\text{Fe}}_{\text{bioaccessible}})/\text{Total Fe}]\times 100\text{;}$$

(2) $$[(\%)\text{Precipitated Fe}]=[(\text{Total Fe}-{\text{Fe}}_{\text{bioaccessible}})/\text{Total Fe}]\times 100\text{;}$$

where Fe_bioaccessible_ represents the fraction of iron released from the bread matrix after the simulated gastrointestinal digestion which remains in solution for the cellular experiments (see below); total Fe is the iron concentration in the final simulated digestion volume and percentages are expressed out of the total iron concentration in the final simulated digestion volume.

### 2.4 Extraction and analysis of inositol phosphates

Inositol phosphates were extracted from 500 mg of freeze-dried ground bread with 20 ml 0.5 M HCl for 3 h at 20 °C under agitation, as reported previously (Skoglund, Carlsson, & Sandberg, 1997). Then samples were centrifuged at 3000*g* for 10 min and the supernatants were kept at −80 °C until used. Quantification of the inositol phosphates was performed by high performance ion chromatography (HPIC) as described previously (Blaabjerg et al., 2010, Phillippy and Bland, 1988). Briefly, prior to the analysis, the samples were centrifuged at 9000*g* for 10 min and the supernatants filtered through 13 mm diameter 0.45 μm pore size polypropylene filters (Kinesis, UK Ltd). Aliquots (50 μl) were injected onto a 3 mm i.d. × 200 mm Carbo Pac PA200 HPLC column (Dionex) fitted with a 3 mm × 50 mm guard column of the same material. The column was eluted at a flow rate of 0.4 ml/min with a gradient of methanesulfonic acid (Acros Organics) delivered from buffers reservoirs containing: A, water; B, 600 mM methanesulfonic acid, according to the following conditions: time (minutes), % B: 0,0; 25,100; 38,100; 39,0; 49,0. The column eluate was mixed using a mixing tee with 0.1% w/v ferric nitrate in 2% w/v perchloric acid delivered at a flow rate of 0.2 ml/min, before passage through a 194 μl volume knitted reaction coil (4 m × 0.25 mm i.d.) obtained from Biotech AB, Sweden. The column, mixing tee and reaction coil were held at 35 °C. Peaks of inositol phosphate were detected at 290 nm in a Jasco UV-2077 Plus UV detector. Chromatographic data were collected from 0 to 49 min and peaks integrated in ChromNav (Jasco) and plotted using GraphPad Prism 5 software. The elution positions of different stereoisomers of different classes of inositol phosphates were determined by the inclusion at regular intervals of a set of standards obtained by extended acid treatment of phytic acid. Inositol phosphates were quantified by reference to the peak area of the standard inositol phosphates.

### 2.5 Food samples and *in vitro* simulated gastrointestinal digestion

The simulated gastrointestinal digestion was performed as described by Glahn, Lee, Yeung, Goldman, and Miller (1998), with minor modifications. Bread samples were first added to 10 ml of pH 2 buffer saline-solution (140 mmol/l NaCl, 5 mmol/l KCl), followed by the addition of pepsin (0.04 g/ml) and incubated for 90 min on a rolling table at 37 °C to simulate gastric conditions. Once the first phase was completed, the pH of the samples was gradually adjusted to pH 5.5, bile (0.007 g/ml) and pancreatin (0.001 g/ml) digestive enzymes were added to the samples, readjusted to pH 7, and incubated for an additional hour on a rolling table at 37 °C to mimic intestinal conditions. At the end of the simulated gastrointestinal digestion, samples were centrifuged at 3000*g* for 10 min and the supernatants used for subsequent cell culture experiments. A volume of 1.5 ml of the digestion was applied to an upper chamber consisting of a Transwell insert fitted with a 15 KDa molecular weight cut-off dialysis membrane (Spectra/Por 7 dialysis tubing, Spectrum laboratories, Europe) suspended over Caco-2 cell monolayers grown in collagen-coated 6 well plates. The bread simulated digestions were incubated on cells for one hour at 37 °C in a humidified incubator containing 5% CO_2_ and 95% air. Inserts were then removed, an additional 1 ml of supplemented MEM was added and cells were incubated for a further 23 h prior to harvesting for ferritin analysis.

*In vitro* digestions were prepared fresh prior to each cell culture experiment. For the studies of iron bioaccessibility, a fraction of the final digestion used for the cellular experiment was collected and stored at −80 °C until analysis by ICP-OES. In addition, prior to the simulated digestions of the breads, ascorbic acid (AA) was added to the gastric digestion (molar ratio of 1:20, Fe:AA) to ensure that all of the iron released during digestion remained in solution. All experiments were performed using the following controls: (a) a blank digestion without any bread samples or added iron to ensure no iron contamination in the *in vitro* digestion/cellular system and (b) a reference digestion of 100 μM of ferrous sulphate heptahydrate (FeSO_4_) solubilised in 0.1 M HCl with 2000 μM of AA. In order to determine how the content of IP6 affects the availability of endogenous iron of the breads and the exogenous iron from a potential mixed-meals, two different types of experiments were performed. Experiments to determine the effects of IP6 content on the bioavailability of endogenous iron in freeze-dried bread powder were conducted using equivalent amounts of iron (50 μM) in each digestion. Experiments to determine the effects of IP6 content of the breads on the bioavailability of exogenous iron were conducted with the addition of 100 μM of FeSO_4_ to the gastric digestion, resulting in a final total Fe concentration of 150 μM.

### 2.6 Cell culture

Caco-2 cells (HTB-37) were obtained from American Type Culture Collection (Manassas, VA, USA) at passage 20 and stored in liquid nitrogen. Cells were grown in Dulbecco’s modified Eagle’s medium (DMEM), supplemented with 25 mM HEPES solution, 10% foetal bovine serum, 1% penicillin (5000 u/ml) and 1% 4 mM l-glutamine (ThermoFisher Scientific, UK) and 1% MEM non-essential amino acids solution (Sigma-Aldrich, UK). Cells were maintained at 37 °C in a humidified incubator containing 5% CO_2_ and 95% air. For iron uptake experiments, Caco-2 cells between passages 30–36 were seeded onto collagen-coated 6-well plates (Bio-Greiner, UK) at a density of 4.75 × 10^5^ cells/well suspended in 2 ml of supplemented DMEMl which was replaced every 2 days. Cells were used on days 13–15 post-seeding according to the method developed by Glahn et al. (1998). In order to ensure a low basal media iron levels, 24 h prior to the initiation of the *in vitro* digestion experiments, the DMEM medium was replaced by Eagle’s minimum essential medium (MEM) without foetal bovine serum supplemented with 10 mmol/l PIPES [piperazine-N,N′ –bis-(2-ethanesulfonic acid)], 26.1 mM NaHCO_3_, 19.4 mmol/l glucose, 1% antibiotic-antimycotic solution, 11 μmol/l hydrocortisone, 0.87 μmol/l insulin, 0.02 μmol/l sodium selenite (Na_2_SeO_3_), 0.05 μmol/l triiodothyronine and 20 μg/l epidermal growth factor (Glahn et al., 1998).

### 2.7 Cell harvest and ferritin analysis

Following 24 h incubation with the bread digestions, the cellular medium was aspirated, the cells were rinsed with 18 Ω MilliQ H_2_O and subsequently lysed by scraping in 200 μl of Cellytic M (Sigma-Aldrich, UK). Cell pellets were kept on ice for 15 min and stored at −80 °C. For analysis, samples were thawed and centrifuged at 14,000*g* for 15 min. Cellular debris was discarded and the supernatant containing the proteins was used for ferritin determination using the Spectro Ferritin ELISA assay (RAMCO, USA). The ferritin concentration in the samples was determined using a microplate reader at an excitation wavelength of 500 nm according to the manufacturer’s protocol. Ferritin concentrations were normalised to total cell protein using the Pierce Protein BCA protein assay (ThermoFisher Scientific, UK).

### 2.8 Statistical analysis

Data are presented as mean values with the standard errors of the means (SEM). The data set was tested for homogeneity of variances by the test of Levene. For multiple comparison, one-way ANOVA followed by a Bonferroni test was used when variances were homogenous or by Tamhane test when variances were not homogenous. Statistical significance was set at P ⩽ 0.05. The statistical analysis was performed using the SPSS package (version 23; SPSS Inc., Chicago, IL, USA).

## 3 Results

### 3.1 Inositol hexakisphosphate was completely degraded by the sourdough bread process

**Fig. 1:**
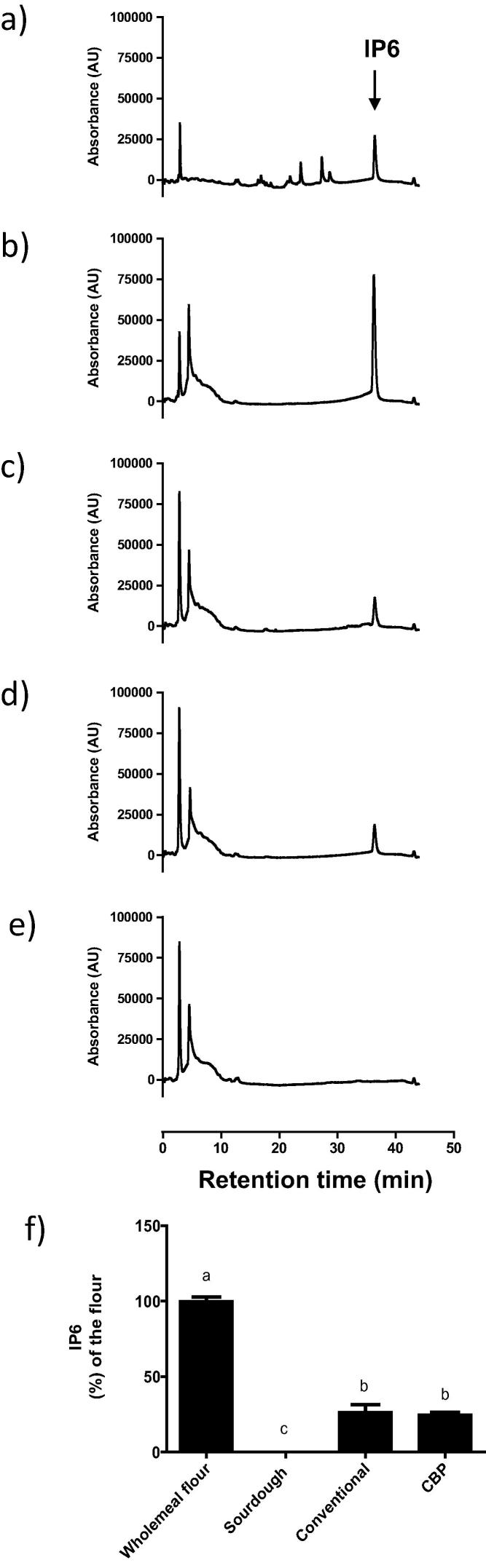
Representative chromatograms (UV 290 nm detection) of the inositol phosphates (IP) of the flour and the different types of bread: (a) Internal standard for different IPs, the arrow denotes IP6, (b) Hereward wholegrain flour, (c) Conventional, (d) CBP and (e) Sourdough bread. (f) Percentage of IP6 in the different bread types relative to the Hereward flour used for bread making. Data represent means ± SEM (n = 4). Means without a common letter differs (P < 0.05).

We investigated the effect of three different commercial bread making processes on the IP6 content of wholegrain bread (Fig. 1). Compared to the wholegrain flour used as the control, the CBP and conventional processes significantly reduced (by about 75%) the concentration of IP6. The reduction was greater with the sourdough process, which completely degraded IP6 from the wholegrain flour. IP6 has a strong chelating iron capacity and the molar ratio of IP6 to iron (IP6:Fe) can be used to estimate its effect on iron absorption (Hurrell & Egli, 2010). We therefore determined the IP6:Fe molar ratio of the IP6 in the breads and their endogenous iron contents (Table 3). This showed that the sourdough process was the only baking process which was able to reduce the ratio to below 1.

### 3.2 Sourdough bread releases endogenous iron during the simulated gastrointestinal digestion

**Fig. 2:**
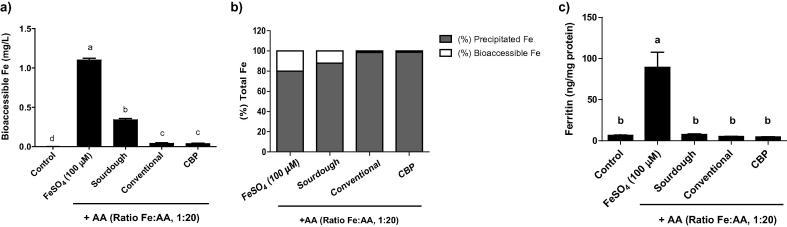
Bioaccessible and bioavailable endogenous iron in the different type of bread supplemented with ascorbic acid (AA) in a molar ratio 1:20 (Fe:AA). (a) Bioaccessible iron in the final gastrointestinal digestion. (b) Percentage of the precipitated and bioaccessible iron fractions in the different bread digestion. (c) Ferritin response in Caco-2 cells exposed to the different gastrointestinal bread digestion. Data represent means ± SEM (n = 6–8). Means without a common letter differs (P < 0.05).

Next we investigated whether the greater reduction of IP6 in the sourdough bread was related to an increase in endogenous iron released during the simulated digestion. As shown in Fig.2a, the sourdough process increased the bioaccesible iron by about 8-fold compared with the other two processes. The percentage of bioaccessible iron increased from the 1.4% and 1.3% of the total iron in the CBP and conventional bread process respectively, to 12% in the sourdough process showing the importance of the reduction of IP6 (Fig.2b).

### 3.3 Endogenous iron released by the sourdough bread does not elicit a ferritin response in Caco-2 cells

In order to determine whether the endogenous iron released by the breads is bioavailable to intestinal cells, we analysed ferritin formation in Caco-2 cells as a measure of cell iron uptake. Caco-2 cells were exposed to different bread digestions, the blank and the positive control (100 μM) (Fig.2c). The levels of ferritin were 6.6 and 89.4 ng/mg of protein for the blank and FeSO_4_ control digest, respectively. Nevertheless, the bread digests did not show a significant increase in ferritin formation compared to the blank control, with 7.8, 5.3 and 4.7 ng/mg of protein for the sourdough, conventional and CBP breads, respectively.

### 3.4 Sourdough bread, but not CBP or conventional breads, results in increased bioaccessibility of added iron in the simulated gastrointestinal digestion

**Fig. 3:**
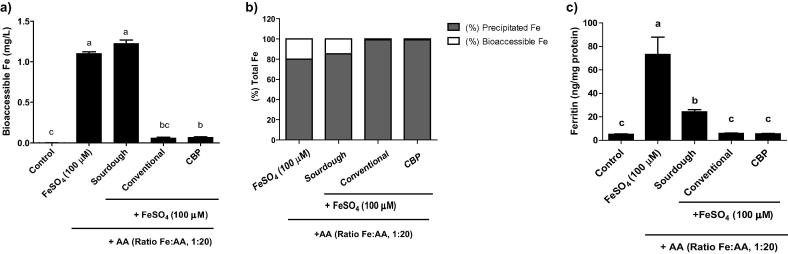
Bioaccessible and bioavailable iron in a simulated mixed-diet adding 100 μM ferrous sulphate and ascorbic acid (AA) in a molar ratio 1:20 (Fe:AA) to the bread prior the gastrointestinal digestion. (a) Bioaccessible iron in the final gastrointestinal digestion. (b) Percentage of the precipitated and bioaccessible iron fractions in the different bread digestion. (c) Ferritin response in Caco-2 cells exposed to the different gastrointestinal bread digestion. Data represent means ± SEM (n = 6–8). Means without a common letter differs (P < 0.05).

We estimated the impact of reduced IP6 content resulting from the different bread processes on the bioaccessibility of exogenous iron by measuring the effect of adding 100 μM of FeSO_4_ to the bread digestion. The addition of exogenous iron significantly increased the quantity of bioaccessible iron in the sourdough bread after digestion, reaching similar values to the positive control (100 μM of FeSO_4_). However, the addition of exogenous iron to the CBP and conventional breads did not increase bioaccessible iron after digestion suggesting that the higher concentration of IP6 in these breads may bind the added iron and limit bioaccessibility (Fig.3a and b).

### 3.5 The reduction of IP6 in the sourdough bread allows for exogenous iron uptake by the Caco-2 cells

Finally, to confirm that the increased soluble iron resulting from the addition of exogenous iron in the sourdough bread digestion is available for absorption by intestinal cells, we measured ferritin formation in the Caco-2 cell model for these bread digestions. As shown in Fig.3c, the sourdough bread with added iron resulted in a significant ferritin increase compared with the blank control (24.3 ng/mg *vs* 5.3 ng/mg) whilst the CBP and conventional breads with added iron did not increase ferritin formation.

## 4 Discussion

This study was performed to compare the effect of three commercial bread making processes on the reduction of phytate in wholemeal flour and to determine iron uptake from these breads after a simulated digestion in the intestinal cells. We further explored the effect of the bread matrix on the absorption of iron from exogenous sources, mimicking a mixed-diet, by the addition of FeSO_4_ to the simulated gastrointestinal digestion of the breads. Our main findings are that: (1) the sourdough bread process degraded the IP6 present in the wholegrain flour, increasing the bioaccessible endogenous iron in the simulated digestion in comparison with the other bread making processes; (2) none of the three types of bread resulted in increasing ferritin formation, a measure of iron uptake, in the Caco-2 cells; (3) the sourdough bread, but not CBP or conventional, allowed an increase in cellular uptake of iron from exogenous sources.

IP6 is degraded during bread making, but the extent can vary extensively depending on the type of bread (flour) and the fermentation process (Brune et al., 1992, Lopez et al., 2001). In the present study, we compared three commercial processes for making wholemeal bread in the UK: the widely used short fermentation CBP and conventional and sourdough long fermentation processes. We observed a 25% greater reduction in the IP6 content of the sourdough bread compared to the conventional and CBP breads. This result is similar to previous studies which reported about 60% reduction in IP6 after the 4 h proving period in sourdough compared with 40% in yeast-fermented dough and in control dough without leavening agent (Leenhardt, Levrat-Verny, Chanliaud, & Remesy, 2005). IP6 has a negative effect on iron absorption, and reducing the molar ratio of IP6:Fe to between 1 and 0.4 has been estimated as the threshold for improving iron bioavailability in cereals (Hurrell & Egli, 2010). However, in general the estimated molar ratio of IP6:Fe for wholegrain bread is higher than 1 (Nielsen, Tetens, & Meyer, 2013), which limits the bioavailability of iron. We found some small differences in the contents of IP6 and iron in comparison with the data reviewed by Nielsen et al. (2013), with a lower IP6 content and higher iron content which probably related to differences in the flour composition and/or bread making processes. However, our phytate contents are similar to those reported in a previous study comparing CBP and a long fermentation (2–3 h) process (Daniels & Fisher, 1981). Similarly, the total iron content in our breads is consistent with Bruggraber et al. (2012), who reported that the iron content of wholemeal bread ranged between 2.4 and 3.4 mg/100 g over the last 70 years. Because of the differences in IP6 and iron contents, we observed a lower molar ratio of IP6:Fe in the CBP and conventional breads than the values reviewed by Nielsen et al. (2013), but in any case, much higher than 1. However, the sourdough process reduced the IP6 content below the limit of detection showing a molar ratio of IP6:Fe below 1, which could explain the 8-fold increase in the bioaccessible iron in the sourdough bread digestion in comparison with the other breads digestion. Despite this increase in the iron released from the sourdough bread digestion, we did not observe an increase in iron uptake by the Caco-2 cells. These findings are in agreement with Sanz-Penella et al. (2012) who reported that a phytase-producing *Bifidobacterium* strain increased bioaccessible iron in bread digestion but had a negligible effect on iron absorption in Caco-2 cells. We therefore suggest that a fraction of the iron released from the bread matrix remains bound and/or encapsulated to other soluble molecules present in the bread digestion, such as dietary fibre, which resist gastrointestinal digestion and reduce its availability for cellular absorption. In fact, it has been recently reported that the mechanical or chemical disruption of the cell walls of the aleurone layer in wheat grain increases ferritin formation in Caco-2 cells after a simulated gastrointestinal digestion (Latunde-Dada et al., 2014). Wholegrain wheat and wholemeal bread also contain ferulic acid, a phenolic compound, which is mainly bound to non-starch polysaccharide (Anson et al., 2009, Li et al., 2008, Lu et al., 2014), and could act as an iron chelator (Chandrasekara and Shahidi, 2011, Teixeira et al., 2013).

Bread is a staple food which can be consumed in combination with other foods or ingredients. It is therefore necessary to determine the effects of different types of bread on the cellular iron uptake from other food sources of iron consumed in mixed meals. We observed that the addition of ferrous sulphate to the sourdough bread increased the bioaccessible iron in the simulated digestion and the iron uptake by the Caco-2 cells, although no increase was detected with the CBP or conventional breads. This demonstrated the effect of the total IP6 degradation in the sourdough bread and the importance of exceeding the critical molar ratio of IP6:Fe to increase the iron bioavailability. However, it is notable that although the amount of bioaccessible iron in the digestion was similar to that in the ferrous sulphate control (without the bread matrix), the ferritin formation was less than 50%. This supports our suggestion that other soluble chelators in the bread matrix, apart from IP6, also restrict iron uptake by the cells. Consistent with our results, Chaoui, Faid, and Belahsen (2006) have reported a similar improvement in the iron status of mice fed a sourdough bread prepared with fortified flour compared to conventional breads prepared with the same flours.

Hurrell and Egli, 2010, Petry et al., 2010, Troesch et al., 2009 have studied the effect of different enhancers and food matrix inhibitors on iron absorption using isotopic Fe labels. They have suggested that the use of a potent phytase enzyme from *Aspergillus niger* in combination with iron fortificants could be a promising approach to improve iron absorption from foods rich in phytic acid and low in iron (Troesch et al., 2009). We have explored this approach *in vitro* using a commercial sourdough bread-making process to reduce the phytate content of the bread, and ferrous sulphate as a fortificant. Our findings are in agreement with those of Hurrell et al. (Troesch et al., 2009) which indicate that reducing the IP6 content in combination with iron fortification can improve iron bioavailability.

In summary, this study demonstrates that the sourdough process leads to the full degradation of IP6 in wholemeal bread and increased the bioavailability of iron in the Caco-2 cell model when the bread was digested in combination with other iron sources, compared with CBP and conventional fermented breads. The wider use of sourdough processes could therefore have significant benefits for iron nutrition.

## Conflict of interest

The authors declare that they have no conflict of interest.

## Figures and Tables

**Table 1:** Amount, yield and iron obtained for each mill fractions from 20 kg of the *Hereward* wheat grain.

Mill fraction	Amount (g)	Yield (%)	Cumulative yield (%)	Fe (ppm)
Break 1	2410	12.0	12.0	8.6
Break 2	1567	7.8	19.9	9.0
Break 3	384	1.9	21.8	21.3
Reduction 1	9343	46.7	68.5	7.8
Reduction 2	1302	6.5	75.0	15.3
Reduction 3	309	1.5	76.6	23.7
Bran flour	198	1.0	77.6	63.4
Offal flour	383	1.9	79.4	68.5
Bran	1905	9.5	89.0	115.8
Offal	1729	8.6	97.6	125.3

**Table 2:** Ingredients (g/kg) employed at the different stages of the three bread making processes.

Bread process		Ingredients	g/kg
Chorleywood		Wholemeal flour	608.23
		Water	367.37
		Ascorbic Acid	0.0625
		Salt	9.0366
		Yeast	12.164
		Bread fat	3.1280

		Wholemeal flour	630.25
	Stage 1 (day 1)	Water	365.54
		Yeast	4.2016

Conventional		Wholemeal flour	435.99
		Water	241.71
	Stage 2 (day 2)	Yeasted 16 h sponge	305.19
		Salt	8.7199
		Yeast	6.2783
		Bread fat	2.0927

		Wholemeal flour	626.11
		Water @ 35 °C	363.14
	Stage 1 (day 1)	Starter culture Lesaffre (LV1)	3.5778
		Salt	7.1556
Sourdough		Wholemeal flour	519.03
		Water	313.49
	Stage 2 (day 2)	Sourdough sponge	155.70
		Salt	7.9584
		Yeast	1.0380
		Bread fat	2.7681

**Table 3:** Endogenous iron, *myo*-inositol hexa*kis*phosphate (IP6) content and the molar ratio IP6:Fe of the three bread processes.

Bread type	Endogenous iron mg/100 g (DW)	IP6 g/100 g (DW)	Molar ratio IP6:Fe
Sourdough	3.2 ± 0.06	*n.d.*	⩽1
Conventional	3.0 ± 0.09	0.19 ± 0.06	5.36 ± 0.97
Chorleywood	2.5 ± 0.07	0.17 ± 0.01	6.01 ± 0.35

Data represent means ± SEM (n = 3). DW, dry weight; *n.d.*, non detected (<0.005 g IP6/100 g).
