# Supplementary material for: Assessment of iron bioavailability from different bread making processes using an in vitro intestinal cell model
Source: Food Chem. 2017 Aug 1;228:91–8. doi: 10.1016/j.foodchem.2017.01.130 (PMC5380216; doi:10.1016/j.foodchem.2017.01.130)
Supplement: Supplementary data 1 [file mmc1.docx]

**Assessment of iron bioavailability from different bread making processes using an *in vitro* intestinal cell model**

Rodriguez-Ramiro I^1^, Brearley CA^2^, Bruggraber SFA^3^, Perfecto A^1^, Shewry P^4^, Fairweather-Tait S^1^.

*^1^Norwich Medical School, University of East Anglia, Norwich, UK*

*^2^School of Biological Sciences, University of East Anglia, Norwich, UK*

*^3^ MRC Human Nutrition Research, Cambridge University, Cambridge, UK*

*^4^Rothamsted Research, Hertfordshire, UK*

**Supplementary tables**

**Supplementary Table S1**

Running conditions used for ICP-OES

| **Analytical Conditions** | **Fe** |
| --- | --- |
| RF power (W) | 1000 |
| Plasma gas (L min^-1^) | 12 |
| Sheath gas (L min^-1^) | 2 |
| Auxiliary gas (L min^-1^) | 0.0 |
| Speed pump (rates min^-1^) | 15 |
| Nebulizer gas flow rate (L/min^-1^) | 0.66 |
| Nebulizer pressure (bar) | 2.77 |

**Supplementary Table S2**

Peak profile measurement parameters

| **Element** | **Fe** |
| --- | --- |
| Wavelength (nm) | 259.9 |
| Number of points | 15 |
| Integration time (s) | 0.5 |
| Increments (nm) | 0.001 |
| Photomultiplicator tube voltage (V) | 935 |
| Photomultiplicator tube gain (%) | 100 |
| Points used | 7 |
| Number of replicates | 3 |
| Calculation mode | Gauss |

**Supplementary Table S3**

Running conditions used for ICP-OES (low flow)

| **Analytical Conditions** | **Fe** |
| --- | --- |
| RF power (W) | 1000 |
| Plasma gas (L min^-1^) | 10 |
| Sheath gas (L min^-1^) | 2 |
| Auxiliary gas (L min^-1^) | 0.0 |
| Speed pump (rates min^-1^) | 10 |
| Nebulizer gas flow rate (L/min^-1^) | 0.73 |
| Nebulizer pressure (bar) | 2.75 |

**Supplementary Figures**


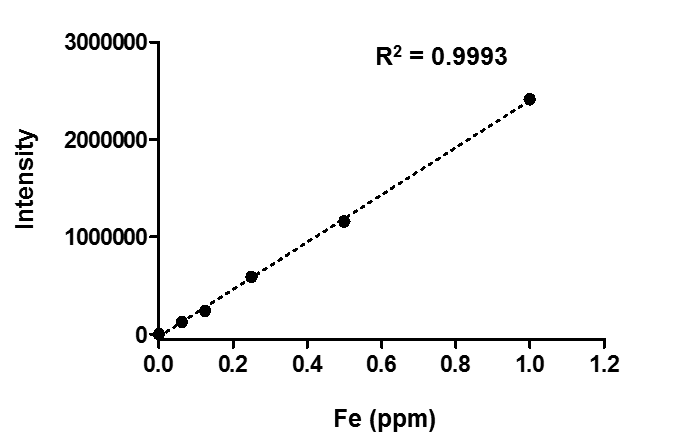


**Supplementary Fig. 1.** Representative calibration curve of the Fe certified standards used to determine Fe in bread, flour and digested bread samples by ICP-OES. A calibration curve was performed for each ICP-OES analysis obtaining in every case a R^2^ > 0.999.
